# Challenges in the Development of Soft Sensors for Bioprocesses: A Critical Review

**DOI:** 10.3389/fbioe.2021.722202

**Published:** 2021-08-20

**Authors:** Vincent Brunner, Manuel Siegl, Dominik Geier, Thomas Becker

**Affiliations:** Chair of Brewing and Beverage Technology, Technical University of Munich, Freising, Germany

**Keywords:** soft sensor, online prediction, bioprocess, multiphase process, data synchronization, sensor fault, fault tolerance

## Abstract

Among the greatest challenges in soft sensor development for bioprocesses are variable process lengths, multiple process phases, and erroneous model inputs due to sensor faults. This review article describes these three challenges and critically discusses the corresponding solution approaches from a data scientist’s perspective. This main part of the article is preceded by an overview of the status quo in the development and application of soft sensors. The scope of this article is mainly the upstream part of bioprocesses, although the solution approaches are in most cases also applicable to the downstream part. Variable process lengths are accounted for by data synchronization techniques such as indicator variables, curve registration, and dynamic time warping. Multiple process phases are partitioned by trajectory or correlation-based phase detection, enabling phase-adaptive modeling. Sensor faults are detected by symptom signals, pattern recognition, or by changing contributions of the corresponding sensor to a process model. According to the current state of the literature, tolerance to sensor faults remains the greatest challenge in soft sensor development, especially in the presence of variable process lengths and multiple process phases.

## Introduction

The biologization of the manufacturing industry is leading to more and more processes that were previously based on chemical synthesis being replaced by biotechnological processes (Buyel et al., 2017). At the same time, the digitalization of these processes is leading to more transparent, lower-risk, and more efficient biological manufacturing (Scheper et al., 2021). At the intersection of these two trends—biologization and digitalization—a multitude of new technologies and approaches have emerged in recent decades. These include, in particular, advances in the fields of data science as well as monitoring and control technology for bioprocesses (Steinwandter et al., 2019). With the introduction of the quality by design (QbD) and process analytical technology (PAT) initiatives, this development has received institutional support (FDA, 2004; Rathore and Winkle, 2009).

Despite advances in bioprocess monitoring, many relevant process variables are still determined offline using laboratory analyses. On this basis, a prediction is made about the expected future behavior of the process. However, this procedure is often not sufficient to effectively react to process changes, for example, through closed-loop control. The development of soft sensors is a remedy to this situation.

A soft sensor (“software sensor”) is a combination of process data (input) and a model that uses these input data to predict a target quantity (output). It is therefore an indirect measurement. The input data used for the prediction are typically composed of signals from hardware sensors and actuators. Dependent on the degree of process knowledge that is implemented, the prediction model can be classified as data-driven, knowledge-based, or hybrid.

The application fields of soft sensors can be distinguished by the nature of the target quantity (Kadlec et al., 2009). The largest application field of soft sensors is the online prediction of physical quantities such as, for example, concentrations of biomass, substrate, intermediate, or product. These types of soft sensors are used when online analyzers are not available or economically feasible for process variables of interest. Further, soft sensors can be used within supervisory control applications to monitor the state of the process on a higher level and detect process faults (Liu et al., 2017; Besenhard et al., 2018; Dumarey et al., 2019). Soft sensors for process monitoring and process fault detection use historical process data to derive higher-level, non-physical process quantities such as latent variables (Kourti, 2005) that indicate deviations from the normal process conditions. Finally, soft sensors can be used to detect sensor faults. The soft sensor here is used to predict the reading of a hardware sensor. A deviation of the prediction and the hardware sensor reading indicates a sensor fault (Brunner et al., 2019). The falsified hardware sensor reading can be reconstructed using the soft sensor’s prediction.

The development of soft sensors poses several challenges to the data scientist. These challenges can be assigned to either the data, information, or knowledge domain. Table 1 lists the most important challenges together with corresponding solution approaches. Most of these solution approaches have been reviewed for the process industry, including phase division (Yao and Gao, 2009), adaption mechanisms for soft sensors (Kadlec et al., 2011), JIT learning (Kano and Fujiwara, 2012; Saptoro, 2014), data synchronization (Ündey et al., 2002), process fault detection (Venkatasubramanian et al., 2003a; Venkatasubramanian et al., 2003b; Venkatasubramanian et al., 2003c), dimension reduction (Pani and Mohanta, 2011), variable selection (Cawley and Talbot, 2010; Souza et al., 2016; Heinze et al., 2018), sensor fault detection and fault tolerance (Isermann, 2006; Isermann, 2011; Das et al., 2012), identification of overfitting (Hawkins, 2004), model maintenance (Wise and Roginski, 2015), digitalization of expert knowledge (Birle et al., 2013), and hybrid modeling (Stosch et al., 2014; Solle et al., 2017).

**TABLE 1:** Overview of the most important challenges and corresponding solution approaches in the development of soft sensors. The challenges are herein broadly assigned to either the data, information, or knowledge domain.

Domain	Challenges	Solution approaches	Details and most important methods
Data	Multiple process phases	Phase detection and division	Algorithms for phase detection can be based on the shape of process trajectories (e.g., sharp peak in specific process variable) or the correlation structure of process variables (e.g., change in loading matrices of latent variable submodels) (Yao and Gao, 2009; Luo et al., 2016)
		Adaption mechanisms	The adaption of the prediction model to multiple process phases can be realized by moving window, recursive adaption, or ensemble-based methods (Kadlec et al., 2011). Just-in-time (JIT) learning is a special case of adaptive modeling, because the local JIT models are built during the online application (Kano and Fujiwara, 2012; Saptoro, 2014)
	Variable process lengths	Data synchronization	Datasets with variable process lengths can be aligned based on: indicator variable techniques, where a measured or computed variable (e.g., maturity index) indicates the progress of the process instead of time; curve registration techniques, where batch trajectories are aligned with respect to process landmarks (Ündey et al., 2002); and dynamic time warping (DTW), where the data patterns are compressed and expanded so that similar features are aligned
	Time-variant and nonlinear behavior	Adaption mechanisms	A prediction model for time-variant data with nonlinear behavior needs to be adaptive rather than static. Adaptive modeling approaches include moving window, recursive adaption, and ensemble-based methods (Kadlec et al., 2011) as well as JIT learning (Kano and Fujiwara, 2012; Saptoro, 2014)
	(Multi)collinearity	Dimension reduction	Latent variable methods (principal component analysis (PCA) or partial least squares (PLS) variants) intrinsically lead to a dimension reduction and thus eliminate (multi)collinearity (Pani and Mohanta, 2011)
		Variable selection	A sound variable selection can reduce (multi)collinearity. Approaches for variable selection include stepwise regression (e.g., backward elimination, forward selection), penalization of model complexity (e.g., based on least absolute shrinkage and selection operator), and through expert knowledge (e.g., a variables’ variance is known to be just due to noise or control error and is thus excluded from model inputs) (Cawley and Talbot, 2010; Souza et al., 2016; Heinze et al., 2018)
Information	Process deviations or faults	Enlarge training data pool	The training data pool can be enlarged by the inclusion of datasets of various fault scenarios and the whole design space instead of only the operating space. Cases that are not covered in the training data pool will lead to unreliable extrapolation of the prediction model
		Process fault detection	Methods of process fault detection can be classified as based on quantitative models, qualitative models and search strategies, and on process history (Venkatasubramanian et al., 2003a; Venkatasubramanian et al., 2003b; Venkatasubramanian et al., 2003c)
	Sensor faults	Sensor fault detection	Sensor faults can be detected via various approaches (Das et al., 2012): symptom signal estimation, where the residual between the original and calculated (predicted) sensor reading indicates a sensor fault (Isermann, 2006; Isermann, 2011); multivariate statistical process control (MSPC), where faults are detected by the contribution of each input variable to underlying statistics of an empirical process model (e.g., PCA or PLS variants); and pattern recognition, where supervised or unsupervised learning algorithms are used to differentiate between faulty and non-faulty sensor data
		Fault tolerance	Fault tolerant soft sensors compensate for faults of inputs to the prediction model by a reconstruction of those inputs (Isermann, 2006; Isermann, 2011). Ensemble-based methods can potentially be used to discard or underweight sub-models with faulty model inputs
	Overfitting	Identification of overfitting	Overfitting can be determined during model evaluation via internal cross-validation (e.g., leave-one-out, k-fold, stratified, or time-series cross validation) and external (holdout) validation (Hawkins, 2004)
		Controlling model complexity	Model complexity can be controlled and thus overfitting can be reduced by a sound variable selection (see above)
	Deterioration of model performance	Model maintenance	In cases where the performance of the prediction model deteriorates due to unseen events (not yet included in the training data pool, e.g., changes in the production strain or seasonal changes in media components), the training data pool and sometimes also the model structure need to be updated (Wise and Roginski, 2015). In all other cases (similar events already included in the training data pool), adaptive modeling approaches such as recursive adaption and ensemble-based methods (Kadlec et al., 2011) as well as JIT learning (Kano and Fujiwara, 2012; Saptoro, 2014) can be used to maintain the prediction model
Knowledge	Implementation of expert knowledge	Digitalization of expert knowledge	Expert knowledge can be digitalized via fuzzy-logic-based approaches in the form of a rule base (Birle et al., 2013) or via first-principle models (Ohadi et al., 2015; Tahir et al., 2019)
		Hybrid modeling	Data-driven modeling can be combined with knowledge-based approaches to make use of available expert knowledge (Stosch et al., 2014; Solle et al., 2017). Hybrid modeling often results in a combination of the advantages and compensation of disadvantages of the two approaches

A small number of these reviews address bioprocesses, but in their majority, they play only a tangential role. Several of the above approaches are equally applicable to bioprocesses (e.g., variable selection, dimensional reduction). However, what needs an updated review or has not yet been reviewed at all in the context of bioprocesses are the following three challenges:

• variable process lengths,
• multiple process phases, and
• sensor faults.

Especially for bioprocesses, these challenges often occur in combination, so that solution approaches are becoming increasingly complex: Sensor faults, which impede the reliability of soft sensors, are more difficult to detect or compensate for in processes with variable lengths and dynamic behavior (Brunner et al., 2019); data synchronization (for processes of variable lengths) is more complex for multiphase processes (Doan and Srinivasan, 2008). The focus of this review is thus on the synchronous consideration of these three challenges of soft sensor development. This review aims to critically evaluate the corresponding solution approaches regarding their practicality and applicability to bioprocesses. The following applies here: As simple as possible, as complex as necessary.

This review article is structured as follows. First, an overview of the status quo in the development and online application of soft sensors is provided. Here, the typical steps of soft sensor development and the state of the art in online implementation are described. The following chapter concerns the challenges in soft sensor development for bioprocesses from a data scientist’s perspective, namely, variable process lengths, multiple process phases, and sensor faults. The corresponding solution approaches are critically discussed. This chapter is followed by a conclusion that reveals the greatest remaining research gaps in soft sensor development for bioprocesses.

## Soft Sensors: The Status Quo

Soft sensors have become an important tool within the QbD/PAT framework, as reviewed by Mandenius and Gustavsson (2015), Randek and Mandenius (2018), and Rathore et al. (2021). One reason is that they are often the only means of determining critical process parameters (CPP) or critical quality attributes (CQA) online at all (Capito et al., 2015; Melcher et al., 2015; Sauer et al., 2019; Spann et al., 2019; Walch et al., 2019; Pais et al., 2020; Wasalathanthri et al., 2020a). Making these quantities measurable by means of soft sensors, in turn, allows CPPs or CQAs to be closed-loop controlled (Birle et al., 2015; Matthews et al., 2016; Voss et al., 2017; Brunner et al., 2020; Gomis-Fons et al., 2020). This type of control, also called inferential control, plays an important role in the automation of bioprocesses, since by far not all process quantities to be closed-loop controlled can be measured directly (Rathore et al., 2021).

As mentioned at the beginning, soft sensors are used to indirectly measure a target variable by combining a predictive model with corresponding input data. Process data used as input to soft sensors can compose differently depending on the organism (bacteria, yeast, filamentous fungi, mammalian or insect cells, etc.) used in upstream processing (USP) and the techniques used in downstream processing (DSP). Instrumentation of bioprocesses and thus possible input data for soft sensors have recently been reviewed by several authors (with varying emphases): Simon et al., 2015 (industrial application); Biechele et al., 2015 (USP, disposable technology); Mandenius and Gustavsson, 2015 (price, utility, and relevance of online analyzers for soft sensor development); Claßen et al., 2017 (spectroscopic sensors); Wasalathanthri et al., 2020b (spectroscopic sensors, chromatography, and mass spectrometry); Gargalo et al., 2020 (spectroscopic sensors, biosensors, and free-floating wireless sensors). Therefore, only a compact selection of the most important process variables and analyzers, respectively, is given in this article. Typical online process data are composed of at least the following readings: flow rates, (differential) pressure (Krippl et al., 2021), temperature, pH, stirrer speed, pO_2_, off-gas CO_2_/O_2_, and conductivity. Often, this standard instrumentation is supplemented by advanced measurement principles, such as turbidity (transmission, transflexion, reflection), impedance, pCO_2_, high performance liquid chromatography (Dumarey et al., 2019), flow cytometry, *in-situ* microscopy, ultrasound, biosensors, proton-transfer-reaction mass spectrometry (Berbegal et al., 2020), and, last but not least, various spectroscopic techniques, such as ultraviolet–visible, near- or mid-infrared (Capito et al., 2015; Sauer et al., 2019; Walch et al., 2019; Wasalathanthri et al., 2020a; Cabaneros Lopez et al., 2021), 2D fluorescence (Melcher et al., 2015; Bayer et al., 2020), Raman (Matthews et al., 2016; Voss et al., 2017), and nuclear magnetic resonance (Kern et al., 2019).

As mentioned, the choice of analyzers used for monitoring and control depends on the used production organism. In mammalian bioprocesses (e.g., Chinese hamster ovary cells), for example, the cell concentration is in most cases significantly lower than in microbial bioprocesses (e.g., *Pichia pastoris*, *Saccharomyces cerevisiae*, *Escherichia coli*). Further, metabolite concentrations, which are particularly relevant in mammalian bioprocesses such as ammonium and lactate (Matthews et al., 2016), are relatively low. Due to higher growth rates, the cultivation time is typically shorter for microbial than for mammalian bioprocesses. For the development of soft sensors, special challenges may therefore arise for the respective expression system: First, the accuracy of the reference and online measurements limits the accuracy of the resulting soft sensors, which can take effect when analyte concentrations are low. Second, faster processes require higher measurement frequency according to the Nyquist–Shannon sampling theorem (microbial: ca. 20–120 h^−1^ (Voss et al., 2017; Cabaneros Lopez et al., 2021); mammalian: ca. 0.5–12 h^−1^ (Ohadi et al., 2015; Matthews et al., 2016)). This must be considered when specifying the prediction frequency of the soft sensor. Especially with the complex preprocessing necessary for spectroscopic data (see next section), the computational power can limit the prediction frequency of the soft sensor (Afseth et al., 2006).

Following this description of possible input data to a soft sensor, the subsequent section shows step by step how to develop a soft sensor. Afterwards, the state of the art in online implementation of soft sensors is shown, i.e., how the soft sensor is concretely used for online prediction.

### Workflow of Soft Sensor Development

The development of soft sensors has been reviewed by several authors. Systematic approaches to soft sensor development have been presented by Fortuna et al. (2007), Kadlec et al. (2009), and Souza et al. (2016) for the process industry and by Haimi et al. (2013) for wastewater treatment plants. They all show a similar workflow. However, the focus of these review articles is on data-driven modeling approaches, and knowledge-based modeling approaches are for the most part neglected. Khatibisepehr et al. (2013) present a systematic workflow for soft sensor development based on Bayesian methods, which inherently combine knowledge-based and data-driven modeling.

**FIGURE 1:**
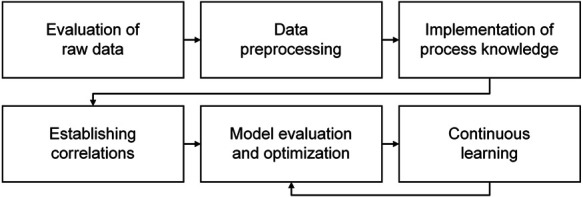
Basic workflow of soft sensor development. A loop exists between model evaluation and optimization and continuous learning; however, revisions of the first four steps will in many cases be necessary to develop a sufficiently accurate and robust soft sensor.

The basic workflow used as a framework in this review article generally assumes a hybrid use of knowledge-based and data-driven approaches (Figure 1). The core of soft sensor development is setting up and evaluating the prediction model. Besides these mandatory steps, the workflow is nonrigid: It depends on the individual case (degree of process knowledge, noisiness of inputs, need for model maintenance, etc.) whether all steps are conducted to the full extent.

The first step in soft sensor development is to evaluate the available raw data in terms of outliers and patterns in the datasets. Outlier analysis is important to identify samples or measurements that distinctly stand out from the rest of the data. An initial correlation analysis between model input and output can provide a matrix of correlation coefficients (e.g., Pearson’s), which helps to assess relationships among the data. When interpreting the results of correlation analysis, however, one must keep in mind that correlation is not equivalent to causality. The correlation analysis can, in combination with available process knowledge, already be employed to preselect information-bearing model inputs (Melcher et al., 2015; Bidar et al., 2018). These analyses provide the basis for the selection of suitable data preprocessing and modeling methods.

The purpose of data preprocessing is to transform the raw input data into a form that minimizes the effect of noise and outliers while preserving the information content. Methods of data preprocessing include formatting, centering, scaling (e.g., to variance), and—specifically for spectroscopic data—baseline correction and peak alignment (Afseth et al., 2006; Matthews et al., 2016; Voss et al., 2017). Signal processing by smoothing and filtering (e.g., Hampel filter (Pearson et al., 2016)) can help to reduce noise and eliminate outliers. However, it is important to note that all preprocessing measures applied during model establishment must also be executable online.

Process knowledge can be implemented into the soft sensor model. Knowledge-based model parts such as first-principle models (Ohadi et al., 2015; Steinwandter et al., 2017; Pappenreiter et al., 2019; Tahir et al., 2019; Krippl et al., 2021) can be employed to develop a more accurate and robust model. Process knowledge in the form of linguistic expressions can be digitalized using approaches based on fuzzy logic, as reviewed by Birle et al. (2013).

After these preceding steps, the actual correlation—the core of the soft sensor algorithm—is established. This correlation model maps the process data X (input) to the target quantity y (output) using model coefficients b. In its simplest, linear form, this model can be formulated as:

y=bX. (1)

If more than one target quantity is predicted with the same model, the vector y in Eq. (1) is replaced by the matrix Y.

Taking into account the application fields of soft sensors described above, y can be a physical quantity that can be measured only offline (online prediction), a higher level, non-physical quantity (process monitoring and process fault detection), or the reading of a hardware sensor (sensor fault detection). Various modeling techniques have so far been applied for soft sensor development, including variants of multiple linear regression (MLR; Jenzsch et al., 2006), partial least squares regression (PLSR; Sokolov et al., 2015; Voss et al., 2017; Zheng and Song, 2018; Walch et al., 2019; Cabaneros Lopez et al., 2021), principal component regression (PCR; Zhu et al., 2018), artificial neural networks (ANN; Paquet-Durand et al., 2017; Zhang et al., 2020), and support vector regression (SVR; Voss et al., 2017; Meng et al., 2019). The choice of the right modeling method depends on the degree of (multi)collinearity, nonlinearity, and the availability of process knowledge.

The model is typically trained, i.e., b is determined, using historical data $X_{\mathrm{hist}}$ and $y_{\mathrm{hist}}$ (exception: just-in-time (JIT) learning), so that $y_{\mathrm{hist}}=bX_{\mathrm{hist}}$. Subsequently, the resulting model needs to be evaluated in terms of goodness-of-fit, predictivity, and robustness (OECD, 2014). Here, the received model is used to predict the target quantity ${\hat{y}}_{\mathrm{hist}}$, so that ${\hat{y}}_{\mathrm{hist}}=bX_{\mathrm{hist}}$. After training and model evaluation, b can be used together with online process data $X_{\mathrm{on}}$ to predict the target quantity ${\hat{y}}_{\mathrm{on}}$, so that ${\hat{y}}_{\mathrm{on}}=bX_{\mathrm{on}}$.

**FIGURE 2:**
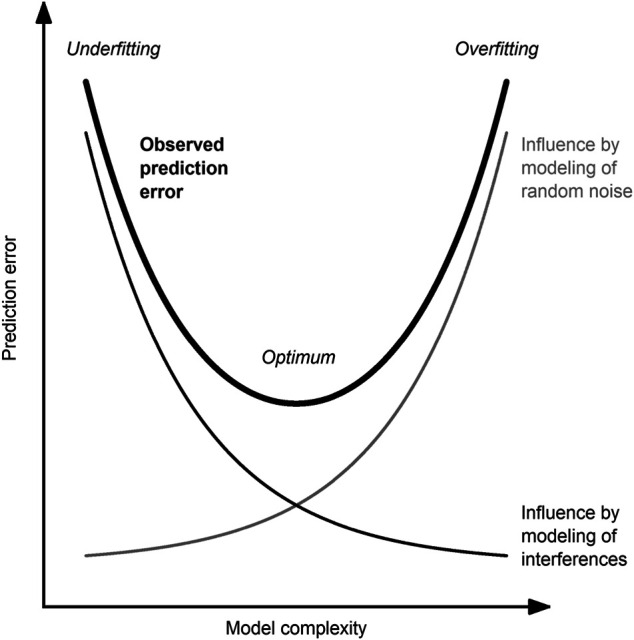
Between the poles of underfitting and overfitting. The observed error (thick, black line) of a predictive model is influenced by the modeling of random noise (undesired; thin, gray line) and interference (desired; thin, black line). The optimal model complexity is a trade-off between these two competing effects and is case-dependent.

For the robustness of the developed model, it is crucial that the model has neither too many nor too few model inputs nor too high nor too low model complexity, respectively (Figure 2). Methods to determine the optimal model complexity have been reviewed by several authors (Cawley and Talbot, 2010; Souza et al., 2016; Heinze et al., 2018).

Even with a robust and sufficiently accurate soft sensor, model quality or prediction performance, respectively, usually deteriorates if the process characteristics change (Kano and Fujiwara, 2012). Therefore, the maintenance or recalibration of soft sensors—just as for hardware sensors—is necessary in practice to preserve the quality of their prediction performance. In this context, model maintenance refers to the (automatic) adaptation of models in the event of changing system conditions. For the prediction models of a soft sensor, this means that the model parameters and, if necessary, the entire model structure (e.g., number and type of input variables) must be adapted over time.

Which programming environment or software solution is used to develop soft sensors in practice? Soft sensor development in the academic environment typically takes place in a programming language of choice such as Matlab (The MathWorks Inc.), Python, or R. The corresponding programming environments provide steadily growing libraries of functions or toolboxes for signal processing, data preprocessing, and model calibration and validation. Especially in the industrial environment, software specially developed for chemometrics is often used for soft sensor development (e.g., SIMCA by Sartorius AG; Unscrambler by Aspen Technology Inc.). Here, the full flexibility of development via program code is exchanged for a relatively straightforward and guided development process. Also, many vendors of online analyzers offer software modules for soft sensor development. In particular, vendors of spectroscopic sensors should be mentioned here (e.g., OPUS suite by Bruker Corp., iC suite by Mettler Toledo Inc., GRAMS suite by Thermo Fisher Scientific Inc.), but also vendors of other multivariate sensors (e.g., BlueVis by BlueSens gas sensors GmbH) offer corresponding software modules. Some software tools (chemometric and analyzer software) also offer the option to embed scripts generated via the above-mentioned programming languages into the soft sensor algorithm. This allows adding customized functions for signal processing and data preprocessing as well as developing prediction models that might not be included in the commercial software tool. Finally, soft sensors can also be developed on cloud-based platforms (e.g., MindSphere by Siemens AG, Predix by General Electric Co.) to have access to a wide variety of data processing and modeling tools and to be able to share the developed soft sensors across plant or company boundaries (Chen et al., 2020; Kabugo et al., 2020).

### Online Implementation of Soft Sensors

How is a soft sensor used in practice for online prediction? In theory, the soft sensor is merely a combination of input data and a prediction model (see definition above). In practice, however, several additional aspects must be considered if a soft sensor is to be used for online prediction, i.e., implemented online.

First of all, online implementation of soft sensors requires at least communication between field (sensors and actuators) and control level (programmable logic controller and/or process control system) and in most cases also supervisory level (supervisory control and data acquisition, SCADA, and/or other data management system). The data used as inputs to the soft sensor can originate from various sources (Steinwandter et al., 2019). Therefore, a standardized communication between these sources and the software instance in which the soft sensor is implemented is essential. While a variety of standard communication protocols exist for communication between field and control level (4–20 mA, Modbus, Profibus, etc.), it is communication via OPC UA (open platform communications unified architecture) that seems to become the predominant standard for communication in the control and supervisory level (Chen et al., 2020; Biermann et al., 2021). Recent efforts even aim at field-level communication using OPA UA (Veichtlbauer et al., 2017). OPC UA, unlike its predecessors of OPC classic (data access, alarms and events, historical data access), allows hardware- and platform-independent communication.

Once the communication and thus the data flow between field, control, and supervisory level has been established, the question arises on which level of the automation pyramid the soft sensor is implemented. Technically, it is possible to implement soft sensors directly in the control level. However, the implementation of scripts directly in the control system is intended for end users only in exceptional cases and the proprietary language must be used (Nair et al., 2020). Systems above the control level, on the other hand, commonly offer the possibility to implement soft sensors directly or indirectly. In the direct variant, soft sensors are implemented in the SCADA (e.g., MFCS by Sartorius AG, Eve by Infors AG, BioXpert by Applikon Biotechnology BV) or other data management system (e.g., SIMATIC SIPAT by Siemens AG, synTQ by Optimal Industrial Technologies Ltd., xPAT by ABB Ltd., Lucullus PIMS by Securecell AG, LabVIEW by National Instruments Corp.). Here, preprocessing steps and model calculations can be implemented directly to a certain extent. More importantly, these software tools often offer the possibility to communicate with external chemometric or analyzer software (Matthews et al., 2016; Voss et al., 2017; Dumarey et al., 2019) or to integrate customized scripts that are executed online (Besenhard et al., 2018). In this indirect variant, soft sensors are implemented in real-time capable chemometric (e.g., SIMCA-online by Sartorius AG (Voss et al., 2017), Process Pulse by Aspen Technology Inc.) or analyzer software (e.g., CMET by Bruker Corp. (Wasalathanthri et al., 2020a), iC Quant by Mettler Toledo Inc. (Wu et al., 2015)) that communicates with the SCADA or data management system. Here, communication often already takes place via OPC UA (Kern et al., 2019). In this indirect implementation, the chemometric or analyzer software preferentially communicates information (e.g., the predicted value) rather than data back to the SCADA or data management system (Luttmann et al., 2012).

The PAT software products mentioned in this section are only a selection and should not be seen as a recommendation. For a more comprehensive overview of PAT software, the reader is referred to Chew and Sharratt (2010). The authors also list whether the respective software is compliant with regulatory requirements for electronic records and signatures according to 21 CFR Part 11 (FDA, 2003).

When soft sensors are implemented in an industrial environment, they must first undergo an intensive functional and risk assessment (qualification). A step-by-step guidance for structured development and implementation has been proposed by Randek and Mandenius (2018). This guidance considers the regulatory validation requirements for software including recommended protocols for installation, operational, and performance qualification. The validation of software, especially in the pharmaceutical environment, commonly follows guidelines such as GAMP 5 (ISPE, 2008), 21 CFR Part 11 (FDA, 2003), or EU GMP Annex 11 (EC, 2010).

## Challenges in Soft Sensor Development for Bioprocesses

This chapter concerns the challenges in soft sensor development for bioprocesses from a data scientist’s perspective, namely, variable process lengths, multiple process phases, and sensor faults. For each of these three challenges, the problem statement is initially outlined. Subsequently, the solution approaches are critically discussed, linking them to the other two challenges, wherever possible. Each solution approach is summarized at the end particularly regarding its practicality and applicability to bioprocesses.

### Variable Process Lengths

#### Problem Statement

From a process engineering perspective, the end of a bioprocess is defined either by the expiration of a certain process time or by the occurrence of a certain process event. Such termination events can be, for example, the reaching of a target value for the biomass or product concentration or a specific pattern in the process data (e.g., a CO_2_ peak indicating the consumption of a carbon source).

In the case of an event-driven process end, the process length can vary from batch to batch due to multiple sources of variance. Besides the typical variance of biological reactions, variance can be introduced by raw materials (e.g., media or feed), by preceding processing units (e.g., preculture), or by deviations in the current process itself.

The variable length of process runs can lead to the following problems. First of all, it can distort the equal weighting of the individual datasets during model development and evaluation if the reference data $y_{\mathrm{hist}}$ are generated at a constant frequency: More reference data points for longer processes lead to an overweighting of longer processes compared to shorter ones. Secondly, in case a dynamic soft sensor model incorporates time as an input variable to compensate for time-variant behavior, variable process lengths lead to another problem: Model performance may deteriorate to the extent that the rate of process progress deviates (i.e., the process is too fast or too slow) from the historical data that were used to train the model. Thirdly, in multiphase processes, the sources of process variance described above can lead to deviations in the time of occurrence of process events (Ündey et al., 2002). If this issue is not accounted for, the adaptation of a soft sensor to process phases could be impeded.

**FIGURE 3:**
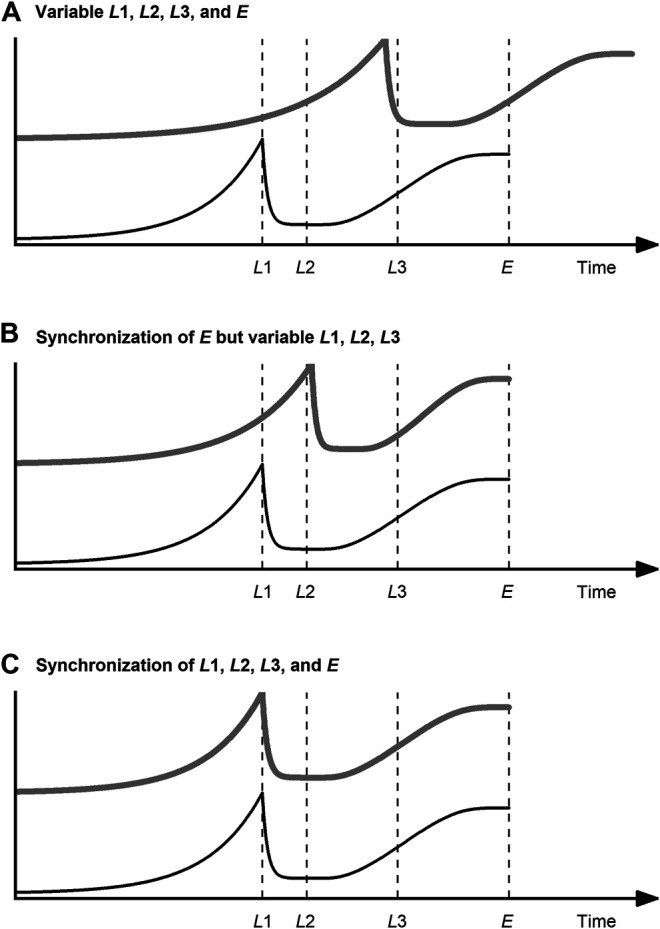
Data synchronization of a query curve (gray, thick line) related to a reference curve (black, thin line) of a fictional process variable with landmarks L1, L2, and L3 and process end E (dashed line): **(A)** Initial situation with variable E and variable L1, L2, and L3; **(B)** Synchronization of E but no coincidence of L1, L2, and L3; **(C)** Synchronization of E, L1, L2, and L3. The landmarks L1, L2, and L3 represent typical curve features, namely a local maximum, a local minimum, and a trend reversal, respectively. The trigger event for this fictional process to end (E) is the reaching of a plateau after L1, L2, and L3 have been reached.

Various methods of data synchronization have been developed to address the challenge of variable process lengths. Data synchronization has two goals, as illustrated in Figure 3 for a fictional process variable: on the one hand, to bring all process datasets to the same lengths (Figure 3B); on the other hand, to ensure that the relevant process events (landmarks) coincide (Figure 3C).

The three techniques used most commonly for data synchronization are discussed in the following: indicator variable, curve registration, and dynamic time warping (DTW). The goal of all these methods is to find a warping function h that replaces the time t on the abscissa and thus to obtain synchronized process data $X_{\mathrm{sync}}$ (Ramsay and Silverman, 2005):

$$X_{\mathrm{sync}}=X\left[h\left(t\right)\right].$$ (2)

As part of soft sensors that are adaptable to variable process lengths, the synchronization algorithm needs to be executable both offline during model development (for $X_{\mathrm{hist}}$ and $y_{\mathrm{hist}}$) as well as during the online application (for $X_{\mathrm{on}}$).

As with all, the choice of the data synchronization method is highly dependent on the process being monitored (Rato et al., 2016; Rato et al., 2018). It should also be noted that, regardless of the method used for data synchronization, all subsequent levels of the monitoring algorithm (soft sensor prediction, fault detection, etc.) depend for better or worse on the robustness and accuracy of the synchronization method used.

#### Indicator Variable Techniques

In this method, the time scale is replaced by an alternative scale, the indicator variable. The indicator variable can be either a real (physical) process variable or an estimated process progress, often referred to as maturity index or percent completion. Process variables that are used as termination criteria for the process or as trigger variables for an automation system are particularly suitable as indicator variables (Ündey et al., 2003; García-Muñoz et al., 2011). Examples of process variables suitable as indicator variable are decrease of substrate concentration (Ündey et al., 2002), cumulative feed volume (Ündey et al., 2003), bioreactor volume, and biomass concentration (Rato et al., 2016). Regardless whether a real process variable or a maturity index is used, the indicator variable should ideally progress strictly monotonically, continuously, and smoothly and have the same start and end value (e.g., 0 and 100 % maturity) for all process runs (Nomikos and MacGregor, 1995; Ündey et al., 2002; Ündey et al., 2003).

When developing a prediction model for the maturity index, the percentage of process progress is calculated for the training data, e.g., by a simple linear transformation. The model requires monotonically progressing variables that correlate with process progress. Examples of the use of a maturity index for data synchronization in bioprocesses can be found in Krause et al. (2015) and Brunner et al. (2019). Both studies demonstrate how a maturity index based on a PLS model can be used to determine process progress online and thus enable adaption to the time-variant behavior of biological batch processes. Only through information about the process maturity was it possible to detect sensor faults in the respective bioprocesses.

Ündey et al. (2003) addressed the challenge of variable process length for a multiphase process, namely a simulated fed-batch penicillin fermentation with two phases (batch and fed-batch phase). They proposed using separate indicator variables for each process phase to compensate for the variable lengths of the phases. As a result, the authors were able to construct tighter control limits for an MSPC model, which in turn enabled faster fault detection. A similar approach was presented by García-Muñoz et al. (2003) for an industrial drying process with three process stages. It was shown that incorporating warping information—i.e., “the information that comes out of an alignment” (García-Muñoz et al., 2003)—resulting from the stage-by-stage alignment can improve a quality prediction model.

**In summary**, indicator variables are suited for data synchronization of bioprocess data under the condition that there is a minimum understanding of the temporal behavior of the process variables. If this knowledge is available and especially if the process variable used as the indicator variable is used as termination criterion for the process, there is no more robust and simple method than this. Problems with the prediction of the maturity index can occur if the input variables of the model change fast in certain process phases and slowly in others. This is not uncommon, especially in USP (lag vs. exponential phase). Even if this is considered by using a non-linear model, the resolution of the input variables restricts the relative accuracy in “slow” process phases. This resolution is determined by the sensors and actuators used. In cases where it is difficult or impossible to find or calculate an indicator variable that comes close to the above-mentioned requirements (strictly monotonically progressing, etc.), curve registration techniques or DTW should be considered. Finally, it must be stressed that indicator variable techniques are per se designed to be independent of any landmarks. These structural features, which are especially helpful for multiphase processes, are ignored during data synchronization and thus cannot be exploited. Data synchronization with indicator variable techniques is therefore limited to the scenario shown in Figure 3B.

#### Curve Registration Techniques

Within functional data analysis, curve registration is referred to as the process of aligning one function curve to another (Ramsay and Silverman, 2005). In this sense, the term curve registration does not differ from the term data synchronization, only that it refers specifically to functional data. The process data are seen as observations of an underlying continuous function (Ündey et al., 2002). The curves are aligned with respect to their structural features, referred to as landmarks. These landmarks can be certain levels, extrema (minima, maxima), or trend reversals (see L1, L2, L3, and E in Figure 3). The relevant landmarks are identified using process knowledge and/or numerical computations, such as first and second derivative, respectively, and zero crossing (Ündey et al., 2002; Ramsay and Silverman, 2005). After matching the landmarks between reference and query, the sections between the landmarks are warped, which in the simplest case means that they are resampled linearly.

Williams et al. (2001) and Ündey et al. (2002) used curve registration to align the process data of a simulated fed-batch penicillin fermentation. For the alignment of multivariate data, the authors suggest first aligning all process data with respect to the landmarks of the most important variable (determined, e.g., via process knowledge). In the second step, a principal component analysis (PCA) is carried out and the process variables are aligned with respect to the landmarks of the first principal component. The second step is repeated until the landmarks converge. In these studies, it was shown that curve registration provides relatively smooth variable trajectories after the alignment compared to DTW; in this way, fewer false alarms occurred with MSPC-based fault detection. In one other of the few examples from the bioprocess field, Andersen and Runger (2012) used landmarks of a pharmaceutical batch fermentation process for data synchronization. The significant landmarks were automatically identified as the zero crossings of a continuous Gaussian wavelet transformation (Bigot, 2006). Afterwards, the resulting curve segments were warped linearly and piecewise for each segment.

**In summary**, curve registration techniques allow not only the alignment of variable lengths—as with an indicator variable—but also the alignment of curve features. Scenario C in Figure 3 can therefore be achieved. Since the features of many process variables occur simultaneously at phase transitions, curve registration techniques are particularly suitable for multiphase processes (Ündey and Çınar, 2002). However, applications of curve registration for bioprocesses are rare. The existence of this niche in the field of bioprocesses can at most be explained by the circumstance that the indicator variable technique is more intuitive and comparatively easy to implement and DTW can be used with less fine tuning.

#### Dynamic Time Warping

DTW, initially developed for speech recognition (Sakoe and Chiba, 1978), was proposed for the synchronization of process data by Kassidas et al. (1998). Since then, it has become one of the most widely used methods for this purpose. Reasons for this are that not only variable process lengths but also landmarks can be aligned using DTW. Scenario C in Figure 3 can therefore be achieved, just as with curve registration. DTW expands, contracts, or translates the time axis of the datasets in such a way that the shape of the variable trajectory is largely preserved, landmarks coincide in time and all datasets have a uniform number of measuring points. The basic sequence of DTW algorithms is as follows: First, the distance matrix (e.g., Euclidean) between the instants of the reference and the query time series is calculated. Then the warping path is searched for that minimizes the sum of distances and at the same time considers several boundary conditions (local, global, endpoint). Using this warping path, the query time series is aligned to the reference time series by expanding, contracting, and translating.

Since its introduction for data synchronization, the original DTW algorithm has been varied in several ways to address issues such as singularities. Singularity in this context refers to the mapping of a single point of the reference time series to multiple points of the query time series or vice versa. Derivative DTW (DDTW) uses local derivatives of the time series instead of raw data and was proposed for overcoming singularities (Keogh and Pazzani, 2001). DDTW compared to DTW tends to align more based on shape rather than magnitude (Spooner et al., 2018). Since numerical derivation often leads to an amplification of noise, a Savitzky-Golay filtering step can be implemented in the DDTW algorithm to make the alignment more robust (Zhang et al., 2013).

For process data that show sections with many successive landmarks (feature-rich) and then again sections with few landmarks (feature-poor), a fixed warping resolution is often not sufficient. Therefore, a dynamic warping resolution was proposed by Gins et al. (2012). This is achieved by a combination of correlation optimized warping (COW; Nielsen et al., 1998; Fransson and Folestad, 2006) for feature-poor and DDTW for feature-rich sections (hybrid DDTW).

The difficulty with the online application of DTW is that a *partially complete* dataset (query) needs to be aligned with a *complete* dataset (reference). This issue was first addressed by Kassidas et al. (1998) and later further elaborated by González-Martínez et al. (2011). In both studies, the endpoint constraint, i.e., that the endpoint of the query must equal the endpoint of the reference, was omitted. This means, however, that the alignment has to be calculated at each sampling point and each time the recent history of the trajectory has to be considered. For this reason, a computationally efficient way of finding the optimal warping path within a moving window was proposed by González-Martínez et al. (2011), referred to as relaxed-greedy time warping (RGTW). Another online application of DTW was presented by Srinivasan and Qian (2007). They used dynamic locus analysis (Srinivasan and Qian, 2006) to identify the best matching signal segment from a reference library by making use of singular points (landmarks) and thus to determine the state of the process. For the actual online warping, a greedy version of the DTW algorithm, referred to as extrapolative time warping (XTW; Srinivasan and Qian, 2005), was used.

González-Martínez et al. (2014) extended the concept of DTW (offline application) and RGTW (online application) to the problem of multiple asynchronisms for a simulated *S. cerevisiae* fermentation. Multiple asynchronism in this context refers to a combination of at least two of the following asynchronism scenarios: variable process length; no coincidence or overlapping of key process events; initial delay or premature termination of a process. The authors proposed a two-step approach in which the asynchronism pattern is firstly detected based on the warping information and secondly batch synchronization is performed based on the detected pattern.

In the standard DTW procedure (univariate DTW), a single representative process variable is used as a reference to align all other process variables. In certain cases, however, univariate DTW can lead to misleading results; this includes, for example, a delayed measurement in a bypass (on-line) or the bioreactor periphery (at-line) compared to the remaining measurements in the bioreactor (in-line). In these cases, multivariate DTW (MDTW) should be considered. Two fundamental variants are distinguished in MDTW (Shokoohi-Yekta et al., 2015): Either DTW is performed separately for each of the process variables j, resulting in j potentially different alignments (“independent” MDTW); or the warping path is determined via a multidimensional p-norm as cost function, whereby multiple process variables are included in the calculation of the distance (“dependent” MDTW). For a review of MDTW, the reader is referred to Moser and Schramm (2019).

**In summary**, DTW and its variants have—at least for simulated data—proven to be well suited for synchronizing bioprocess data, both offline and online. No process knowledge is necessary to develop this preprocessing method. When dealing with multiple process phases, DTW can be used in two different ways: first, it can be used to detect process phases (Gollmer and Posten, 1996); second, it can be used to align data within a process phase (Doan and Srinivasan, 2008; Spooner et al., 2018). The use of DTW for these purposes is further described in the following section. Finally, it should be noted that the warping information can be used for the classification of deviations from normal operating conditions, such as sensor faults (González-Martínez et al., 2013). However, in order to identify the deviating sensor, each fault scenario of interest must explicitly be included in the training data pool.

### Multiple Process Phases

#### Problem Statement

From a monitoring perspective, industrial processes can take place either in multiple processing units (multistage) or in a single one. A process with a single processing unit (e.g., USP in a bioreactor) can have multiple operational regimes, such as a batch and fed-batch phase, and is referred to as multiphase process. Multiphase processes are often treated analogously to multistage processes (Yao and Gao, 2009), i.e., different process phases are treated as if they took place in separate processing units.

The necessity of considering multiple process phases when developing a soft sensor is obvious: The relationships within the input data X (multicollinearity) and between the input data and the target variable y (correlation) can vary substantially in the individual phases. The challenges discussed in this section refer to changes in the relationships from X to y that are related to the process strategy. These include, for example, an induced change in media composition due to feeding, the start or end of a starvation phase, long-term changes between oxygen-limited and non-limited process conditions, or changes in the temperature setpoint. Changes in the relationships of X to y that are associated with time-variant and nonlinear behavior are not within the scope of this review article, although the respective adaption mechanisms partly overlap. These adaption mechanisms have been excellently reviewed by Kadlec et al. (2011).

Only with much greater development effort or available process knowledge will a global process model attain the same accuracy and robustness as several submodels for each process phase. Graphically expressed, the required model complexity (cf. Figure 2) of a global model is allocated to several less complex local models. This in turn can make it easier to optimize the model (Jin et al., 2015), for example, in terms of avoidance of overfitting.

The main difference between datasets of multistage and multiphase processes is that in multiphase processes the individual phase segments must first be identified and often cannot be precisely separated. The actual modeling step is therefore often preceded by a phase detection and division step. The detection and division is based either on trajectories of phase-sensitive process variables or on the changing correlation structure among the process variables (Luo et al., 2016).

#### Trajectory-Based Phase Detection and Division

The sequence of the most biotechnological processes is not given by nature, but by process experts. Therefore, if knowledge about the process sequence is available, it is reasonable to use it for phase detection and division. The definition of landmarks by process experts leads to a solution that is both robust and comprehensible. An example of this can be found in Spooner et al. (2018), who, in contrast to their previous study (Spooner et al., 2017), first divided a bacterial fermentation process into two phases and then aligned the process data of these phases separately via DTW and DDTW, respectively. The pH and pH correction agent (flow and cumulative amount) signals were used to distinguish the phases. Brunner et al. (2020) used the off-gas CO_2_ signal to detect the consumption of the carbon source and thus the end of the batch phase in a *P. pastoris* fed-batch bioprocess. To make the detection of this landmark (CO_2_ peak) more robust, a threshold for the cumulative amount of pH correction agent was additionally implemented in the phase detection algorithm.

The role of DTW for data synchronization has already been described in the previous section. As a means of detecting process phases, it was first proposed by Gollmer and Posten (1996) for time-varying fed-batch bioprocesses (*E. coli* and *S. cerevisiae*). With the use of historical time trajectories of CO_2_ and O_2_ together with available process knowledge, six different process phases were classified. This reference (prototype) was used in the online application by the DTW algorithm to assign unknown process data to this pattern and thus detect the previously defined process phases. Doan and Srinivasan (2008) proposed a variant of DTW augmented by singular points (landmarks) for the combined detection and synchronization of process phases. They used substrate feed and pH as key variables for the detection of process phases of a simulated fed-batch penicillin fermentation. Phase changes were considered equivalent to the occurrence of singular points and were identified using the methods described in the previous section (Srinivasan and Qian, 2005; Srinivasan and Qian, 2007). DTW (offline) and XTW (online), respectively, were then used for data synchronization within the phase segments.

Especially in USP, phase transitions must be considered, as biological systems involve living cells, which do not react instantaneously to environmental changes. Luo et al. (2016) proposed a framework for adapting process models to a sequence of multiple process phases while explicitly considering phase transitions in a simulated fed-batch penicillin fermentation. They used fuzzy c-means (FCM) clustering for phase detection and division to account for the gradual transition from one steady phase to another. The FCM clustering algorithm was constrained by the temporal sequence of the dataset. Phase-based multiway PLS models were used for prediction in the steady phases, and JIT-PLS models were used during the transition phases.

**In summary**, trajectory-based algorithms are suitable for phase detection and division in cases where a minimum of process knowledge is available. This knowledge is necessary to select the phase-sensitive process variables. Provided that suitable phase-sensitive process variables can be identified, this approach is more comprehensible than the correlation-based approach. This is especially due to the fact that the identified phases usually correspond to operational phases (Yao and Gao, 2009). Finally, it must be emphasized that DTW is suitable not only for data synchronization in the case of variable process lengths, as described above, but also for the detection of multiple process phases.

#### Correlation-Based Phase Detection and Division

A difficulty with the methods mentioned so far is to find variables that are measurable and sensitive to the individual phases (Luo et al., 2016) and whose trajectories are reproducible and as noise-free as possible. In the following, methods are presented that accomplish phase detection and division without the need for process knowledge. These methods are based on changes in the correlation structure among process variables.

Camacho et al. (2008) proposed an algorithm based on latent variable models (PCA or PLS) for the detection and division of process phases for a *S. cerevisiae* and a wastewater treatment process. The whole process dataset is iteratively divided into incrementally smaller phases. At each iteration step, the separation point that leads to the maximum improvement of the explained variance of the PCA or PLS submodel, respectively, compared to the undivided dataset is identified (Camacho and Picó, 2006).

Another method to make use of changes in the correlation structure is to first determine the loading matrices of PCA or PLS submodels following a moving window approach and then to find groups in which the underlying variable correlation remains similar (Lu et al., 2004; Lu and Gao, 2005). These groups can, for example, be determined by k-means clustering (Lu et al., 2004).

However, if different operation modes with variable phase and process lengths are to be considered, the classical moving window approach leads to misleading results. The reason for this is that in the online application it is not clear whether the current moving window coincides with that of the reference (historical data). Therefore, the described method for phase detection and division (Lu et al., 2004) was extended by the ability to identify the current mode of operation (resulting in variable lengths) online. Zhang et al. (2018) generated a series of moving windows within a constrained searching range around the current sample. They then used the k-nearest neighbor rule to identify the most similar time slices. The time slices found in this way are then used as described above (loading matrices of submodels, k-means clustering) to enable online phase detection despite variable phase and process lengths.

Finally, Gaussian mixture models (GMM) have proven to be suitable for phase detection and division for a simulated fed-batch penicillin fermentation (Yu and Qin, 2009; Yu et al., 2013). Here, each phase is represented by a Gaussian component with distinct mean and covariance. The posterior probability is used to group the process data into separate process phases. This concept was later adopted for a real industrial bioprocess (Mei et al., 2017).

**In summary**, for correlation-based phase division, the reduced effort in implementing process knowledge is compensated with an increased effort in modeling. Depending on the modeling method, however, an entirely different division of the process phases may result and fine-tuning of the latent variable models is necessary (Luo et al., 2016). Because the correlation structure is of multivariate nature, the interpretability of the results of the phase division is limited in contrast to most trajectory-based methods.

### Sensor Faults

#### Problem Statement

Sensor faults are defined as deviations of the observed sensor reading from the true value (Balaban et al., 2009; Sharma et al., 2010). They are distinguished according to the type of occurrence as abrupt (stepwise) or incipient (driftwise) faults (Isermann, 2006) and according to the shape of the deviation as bias, precision degradation, and complete failure.

During the training phase of soft sensor development, sensor faults can severely affect the resulting goodness-of-fit and predictivity. If sensor faults are present in the training data $X_{\mathrm{hist}}$ and $y_{\mathrm{hist}}$, these deviations may be reflected in the model coefficient b and the prediction ${\hat{y}}_{\mathrm{hist}}$. In this case, evaluation criteria for goodness-of-fit and predictivity (e.g., $R^{2}$, root mean squared error) are affected. During the online application of the soft sensor, i.e., the prediction phase, sensor faults in $X_{\mathrm{on}}$ may directly affect the prediction of the target quantity ${\hat{y}}_{\mathrm{on}}$.

The validation of sensor readings prior to their use for quality control, e.g., via soft sensors, is therefore of crucial importance, as outlined by Feital and Pinto (2015). A sensor reading is valid if there are no sensor faults or unconsidered influences on the measurement, which can occur due to cross-sensitivity to matrix compounds (matrix effects). Deviations between the observed sensor reading and the true value thus need to be detected, and a decision logic needs to classify the observed sensor reading as reliable (valid) or faulty (invalid). Valid sensor readings can be used for quality control by means of soft sensors, while invalid ones can lead to misleading results.

The fault tolerance of soft sensors, or, in other words, a reliable soft sensor prediction in the presence of sensor faults represents one of the remaining core challenges in the development of soft sensors. The reason for this is that the detection and subsequent compensation of sensor faults alone are difficult to realize, but they become even more complex when the conditions described above (variable process lengths and multiple process phases) occur simultaneously.

This section first discusses various methods of detecting sensor faults. Afterwards, the approaches for tolerance of soft sensors towards sensor faults are discussed.

#### Sensor Fault Detection

**FIGURE 4:**
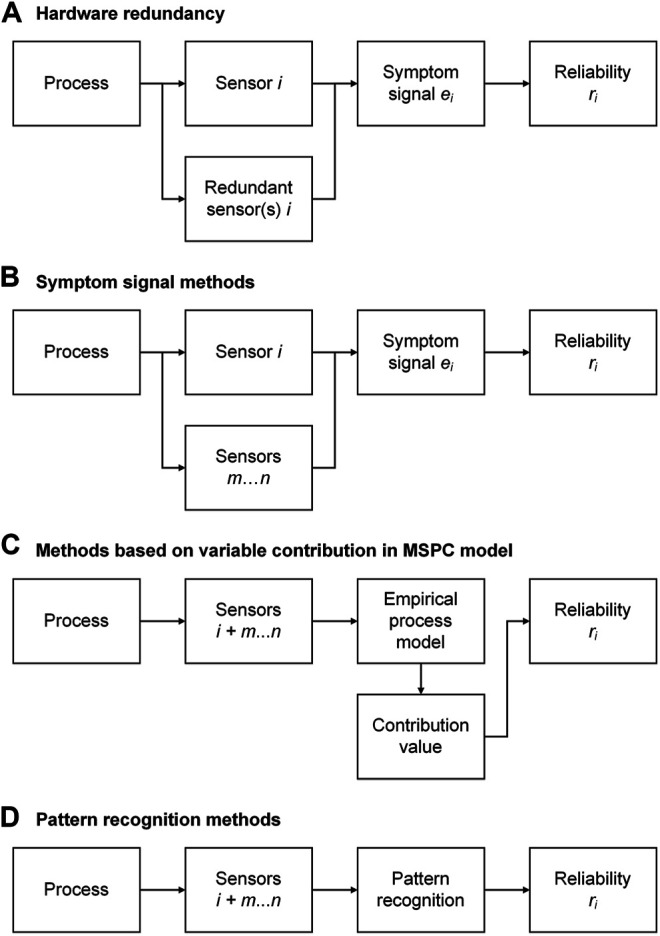
Approaches for sensor fault detection: **(A)** hardware redundancy; **(B)** symptom signal methods; **(C)** methods based on variable contribution in MSPC model; **(D)** pattern recognition methods. The index i refers to *one* type of sensor for *one* physical or chemical variable, whereas m…n refers to *any other* types of sensors. For example, in **(A)**, the reliability of one turbidity sensor (sensor i) is determined using other turbidity sensors in the same bioreactor (redundant sensors i). In **(B)**, the reliability of a turbidity sensor (sensor i) is determined using other sensors such as exhaust gas, dissolved oxygen, and pH (sensors m…n). In **(C,D)**, the entire process data matrix consisting of the turbidity sensor to be monitored (sensor i) and the other sensors (m…n) is used as input to the empirical process model **(C)** and pattern recognition **(D)**, respectively. Approaches **(B–D)** are based on analytical redundancy.

When a sensor i that is used to monitor a bioprocess gives faulty readings, its reliability $r_{i}$ decreases. The aim of sensor fault detection is to detect these faulty readings and thus indirectly determine $r_{i}$. Figure 4 shows four fundamental approaches to sensor fault detection.

Hardware redundancy uses multiple identical sensors to derive the occurrence and size of sensor faults in case of a significant discrepancy among these sensors (as, for example, in airplanes). Voter structures can be implemented into the fault detection algorithm to allow a “democratic” decision on which of the individual sensor values is faulty. If, for example, two of three sensors give a similar reading and the third reading deviates significantly, the third sensor is considered to be faulty. For hardware redundancy, the spatial distribution of the sensors must be considered, and the costs of the sensors limit this approach (Stork and Kowalski, 1999). Hardware redundancy can also be used to determine the type of fault as bias, gain, precision degradation, complete failure, and noise (Kullaa, 2013).

The other three approaches are based on analytical redundancy and are described in the following.

##### Symptom Signal Methods

As mentioned in the introduction, soft sensors themselves can be used to assist in sensor fault detection. Here, the target quantity y of the soft sensor is the reading of the hardware sensor to be monitored. A deviation of the prediction ${\hat{y}}_{\mathrm{on}}$ from the original reading $y_{\mathrm{on}}$ beyond a defined threshold value indicates a sensor fault. The residual between ${\hat{y}}_{\mathrm{on}}$ and $y_{\mathrm{on}}$ is referred to as symptom signal e (Zarei and Shokri, 2014) and can in its simplest form be formulated as:

$$e=y_{\mathrm{on}}-{\hat{y}}_{\mathrm{on}}.$$ (3)

Several authors make use of state-space models for the generation of the symptom signal, as described in the following. Zarei and Shokri (2014) used a nonlinear unknown input observer to generate symptom signals and thereby to detect sensor faults in a simulated continuous stirred-tank reactor (CSTR) process. Alag et al. (2001) proposed a framework for sensor fault detection based on the symptom signal method exemplarily for a gas turbine power plant. They proposed a multi-step algorithm for determining ${\hat{y}}_{\mathrm{on}}$ followed by the generation and evaluation of the symptom signal. First, a redundant prediction for each sensor in the network is generated based on regression methods such as neural networks (redundancy creation). Subsequently, these predictions are fused with original sensor readings into a state-space model based on a Kalman filter approach (state prediction and fusion). The statistical properties of the symptom signal are in combination with probabilistic reasoning finally used to identify both abrupt and incipient sensor faults. Since a symptom signal is created for each sensor, the proposed methodology is capable of detecting multiple sensor faults simultaneously.

Autoassociative neural networks (AANN) were first introduced by Kramer (1991) for sensor fault detection and reconstruction in a simulated chemical batch process. They have proven to be effective in detecting sensor faults in a fermentation process (*Streptomyces virginiae*) with variable process length and multiple process phases (Huang et al., 2002). AANN are feed-forward neural networks consisting of an input, an output, and three hidden layers (mapping, bottleneck, and demapping layer). The outputs of the bottleneck layer are considered equivalent to the principal components of a nonlinear PCA (Kramer, 1991). The key concept of AANN is that the model is trained with fault-free process data $X_{\mathrm{hist}}$ both as input and output, so that $X_{\mathrm{hist}}=Y_{\mathrm{hist}}$. The resulting nonlinear model is used for determining online predictions of the process data, ${\hat{Y}}_{\mathrm{on}}$, based on online measured process data, $X_{\mathrm{on}}=Y_{\mathrm{on}}$; then, analogous to Eq. (3), the residual is calculated for each variable and used for the detection of sensor faults. This concept was extended to a complex nonlinear system with time-delays, namely a multicomponent distillation column (Perla et al., 2004).

The symptom signal method was used by Brunner et al. (2019) to detect sensor faults in a *P. pastoris* batch process. Due to the time-variant behavior and variable lengths of the batch processes, an indicator variable (maturity index) is introduced to predict the process progress online. For each process section, a set of prediction models for ${\hat{y}}_{\mathrm{on}}$ is generated. A regularization approach based on binary particle swarm optimization (PSO) is used to select the 25 best prediction models. The distribution of the predictions ${\hat{y}}_{\mathrm{on}}$ is compared to a moving window distribution of $y_{\mathrm{on}}$ using the Kullback–Leibler divergence (Kullback and Leibler, 1951). The divergence between ${\hat{y}}_{\mathrm{on}}$ and $y_{\mathrm{on}}$ indicates a sensor fault and is used to quantify the sensor reliability $r_{i}$.

Most studies use a fixed threshold for fault detection based on symptom signals. This can lead to false alarms when unforeseen events or noise occur in the sensor network data. In these cases, a time-varying as opposed to a fixed threshold can increase robustness and minimize the fault detection time, as shown by Armaou and Demetriou (2008) for simulated chemical processes. However, if process lengths vary, the threshold needs to adapt dynamically to process progress and not just to process time. For this reason, Brunner et al. (2019) proposed a dynamic threshold, which is calculated by means of the confidence width of $\;{\hat{y}}_{\mathrm{on}}$, which in turn is dependent on the process progress.

In addition to the mere detection of a sensor fault, information about the type of fault may also be necessary for the potential subsequent compensation (fault tolerance). To determine the type of fault as either bias, complete failure, drifting, or precision degradation, Dunia et al. (1996) developed a concept in which the symptom signal is generated using a PCA prediction model.

**In summary**, symptom signal methods are well suited for the detection of sensor faults and they are relatively intuitive due to their similarity to hardware redundancy. The main bottleneck of this approach is the model for the prediction of ${\hat{y}}_{\mathrm{on}}$, which is used for generating the symptom signal. For most bioprocesses, the model needs to consider time-variant behavior and variable process lengths. With the exception of AANNs, this model must be developed separately for each sensor to be monitored. The main advantage of the symptom signal method is that there is a direct reconstruction for the faulty sensor value available. Soft sensors or control systems, which depend on a reliable sensor input, can fall back on the reconstructed value and thus be designed fault tolerant.

##### Methods Based on Variable Contribution in Multivariate Statistical Process Control Model

Multivariate statistical process control (MSPC) and its corresponding empirical process models and control charts are another method to detect sensor faults. The original idea of MSPC is to map a wealth of process data X to one or a few higher-level, non-physical process quantities y or Y, respectively, such as latent variables (Kourti, 2005). Deviations between historical, $X_{\mathrm{hist}}$, and online process data $X_{\mathrm{on}}$ are detected using control charts based on the complementary SPE (squared prediction error; sometimes denoted as Q) and Hotelling’s $T^{2}$ statistics (Nomikos and MacGregor, 1995; Liu et al., 2017; Sánchez-Fernández et al., 2018). Once these test statistics indicate a significant deviation, the contribution of each input variable in $X_{\mathrm{on}}$ to the test statistic(s) is calculated. Sensors or variables, respectively, with a significantly high contribution to the test statistic(s) are associated with a sensor fault. A general analysis of the variable contribution approach is given by Qin (2003). Various methods for decomposing the test statistics to contributions, such as complete, partial, or reconstruction-based decomposition, were analyzed by Alcala and Qin (2011).

Sánchez-Fernández et al. (2018) combined the symptom signal and the contribution-based method for the detection of process and sensor faults. Residuals between predictions and observations for each variable in $X_{\mathrm{on}}$ are used as inputs to a PCA-based MSPC model. Residuals that are calculated with faultless training data are used for calculating the thresholds (control chart limits) for fault detection based on $T^{2}$ and SPE statistics. Multivariate and univariate exponentially weighted moving average control charts are used for the detection of process and sensor faults, respectively. Two simulated benchmark processes (Tennessee Eastman process and wastewater treatment plant) were used for validating the concept.

Another combination of the symptom signal and the contribution-based method was proposed by Yoo and Lee (2006) for sensor fault detection. Here, contribution plots assist in identifying faulty variables. In case the contribution plots indicate a fault, the original measurement is compared with a prediction based on a fuzzy PLS model of the corresponding variable. However, no algorithm was presented on how to derive the sensor reliability or the fault magnitude and type, respectively. The concept was evaluated on a real and a simulated wastewater treatment plant.

Reconstruction-based contributions (RBC) were proposed for sensor fault detection by Yue and Qin (2001). Here, $T^{2}$ and SPE are combined in a fault detection index φ. This combined index proved to have better detectability both for single and multiple sensor faults than if the contributions to $T^{2}$ and SPE are considered separately (Yue and Qin, 2001; Alcala and Qin, 2009). This concept was adopted by Tôrres et al. (2018) for pharmaceutical tablet manufacturing. The RBC approach was extended by Mnassri and Ouladsine (2015) to handle multiple and more complex sensor faults.

A contribution-based approach to sensor fault detection and tolerance was developed by Krause et al. (2015) for the monitoring of a yeast fermentation process. This approach does not consider the contribution to the test statistics as described above, but the direct contribution to the model b and the prediction ${\hat{y}}_{\mathrm{on}}$, respectively, for fault detection. They developed a PLS-based MSPC model using an indicator variable to compensate for variable process lengths. For each process section, a set of MSPC models is generated and PSO is used for finding the best models with respect to historical process data (with normal process behavior). Variable importance in the projection (VIP) scores (Chong and Jun, 2005) were used to evaluate the input variables for their contribution (information content) to the MSPC model. A reduction of the VIP score of a variable is assigned to a fault of the corresponding sensor.

A problem not to be underestimated in contribution-based fault detection is the smearing effect (Alcala and Qin, 2011; van den Kerkhof et al., 2013). Smearing here refers to the “influence of faulty variables on the contributions of non-faulty variables” (van den Kerkhof et al., 2013). Faulty variables (i.e., soft sensor inputs) can thus be concealed, and non-faulty variables can be incorrectly associated with faults. In contribution-based fault detection, groups of correlating variables are often displayed as faulty due to the smearing effect (van den Kerkhof et al., 2013); this is an obstacle especially for the often multicollinear data of bioprocesses.

To account for the nonlinearity of CSTR processes, several authors introduced the kernel PCA (Schölkopf et al., 1998) as a nonlinear extension of the PCA and adapted the calculation of the contributions to the $T^{2}$ and SPE statistics accordingly (Cho et al., 2005; Choi et al., 2005; Alcala and Qin, 2010).

The functional principle of AANN has already been described above for fault detection using symptom signals. Ren et al. (2018) proposed a reconstruction-based AANN to detect faults in nonlinear processes (simulated gas turbine). Both single and multiple faults could be detected despite the occurrence of smearing effects. It was further shown that in this case reconstruction-based AANN is superior to the other investigated methods (contribution plots-based PCA, contribution plots-based AANN, and reconstruction-based PCA) in terms of detection rate.

**In summary**, methods based on variable contribution currently represent the largest share among studies on sensor fault detection in the process industry. The main advantage of these methods is that the MSPC model can be used both for process and sensor fault detection. With only one MSPC model it is theoretically possible to monitor all input variables or sensors, respectively. To the best of our knowledge, however, there is only one study (Krause et al., 2015) that shows that, for highly multicollinear bioprocess data, smearing effects do not prevent successful sensor fault detection. For multiphase processes with variable process lengths, the MSPC models used for defect detection can be developed separately for each phase and a phase-specific indicator variable can be used for time synchronization (Ündey et al., 2003).

##### Pattern Recognition Methods

Unsupervised (clustering) and supervised (classification and regression) pattern recognition has been applied extensively for bioprocess monitoring (Lourenço et al., 2012; Rodríguez-Méndez et al., 2016). Also, in the detection of sensor faults by pattern recognition, a distinction is made between unsupervised and supervised methods.

In the case of unsupervised pattern recognition, the training data consist of fault-free process data. The relationships within the process variables are learned as patterns. A specific deviation from the fault-free pattern can then be assigned to a specific sensor fault (Barbariol et al., 2020). In this context, unsupervised pattern recognition is comparable to the aforementioned methods based on variable contribution in MSPC models: First, a deviation from the fault-free standard process is detected and then it is examined to determine to which variable the fault can be traced. These two approaches (MSPC vs. unsupervised pattern recognition) differ less by this underlying principle than by the modeling methods used (empirical process model vs. clustering algorithm).

Barbariol et al. (2020) used unsupervised anomaly detection algorithms to detect faults of a multiphase flow meter. Artificial faults were added to data of normal operating conditions. The type of fault was identified as either complete failure, bias, precision degradation, or drift by a root cause analysis algorithm.

In the case of supervised pattern recognition, the training data contain sensor faults. These sensor faults can be artificial or real, but in any case, they must be labeled according to their reliability $r_{i}$ or—conversely—their degree of faultiness (1 – $r_{i}$). Faultiness is indicated either binarily (fault = true/false) or on a discrete (Mehranbod et al., 2003) or continuous scale (Guo and Nurre, 1991). Detecting sensor faults becomes a classification problem in case of binary or discrete faultiness and a regression problem in case of continuous faultiness. In both cases, labeled faulty data $X_{\mathrm{hist}}$ represent the inputs and the degree of faultiness (or the converse: $r_{i}$) represents the output. These input and output data are used for training the classification or regression model.

Guo and Nurre (1991) used supervised pattern recognition to detect and reconstruct sensor faults in a space shuttle main engine. Artificial random Gaussian noise was added to parts of fault-free data from normal operation. If the resulting artificial sensor readings are within the valid range, they are assigned a reliability of 0.9; if they are outside the valid range, they are assigned a reliability of 0.1. A feedforward ANN is trained with the manipulated sensor readings as inputs and the corresponding labeled reliability as outputs using a backpropagation algorithm to adjust the weights. In this way, even with a very small amount of original data, sensors whose readings do not match the rest of the sensor network can be identified. It was further shown that supervised pattern recognition is also suitable for the detection of multiple simultaneous sensor faults (Palmé et al., 2011) even in the presence of system failures (Romesis and Mathioudakis, 2003; Mathioudakis and Romessis, 2004). In this case, the training data must cover each of these cases (deviation from normal operation and multiple sensor faults), which causes the number of training patterns to increase rapidly.

In addition to the mere detection of faults, Mehranbod et al. (2003) distinguished between three different fault types (bias, drift, or noise) by identifying fault patterns in a moving window. They trained a Bayesian belief network to detect both single and multiple sensor faults in a polymerization reactor. This concept was later extended for the time-variant behavior of transient processes (Mehranbod et al., 2005).

**In summary**, pattern recognition methods are particularly attractive because ready-to-use—and in many toolboxes also auto-tuned—algorithms of machine learning can be applied to the problem of sensor fault detection without extensive statistical knowledge. At least in mechanical or chemical processes, efficient sensor fault detection can be realized with only little original data from normal operation together with artificial faults (Guo and Nurre, 1991). Despite this high potential, there are, to our knowledge, no studies that have explicitly used the previously trained pattern of sensor faults for their subsequent detection in bioprocesses. This lack of studies is all the more remarkable as pattern recognition methods are particularly efficient with such a high degree of multicollinearity as in bioprocesses.

#### Sensor Fault Tolerance

**FIGURE 5:**
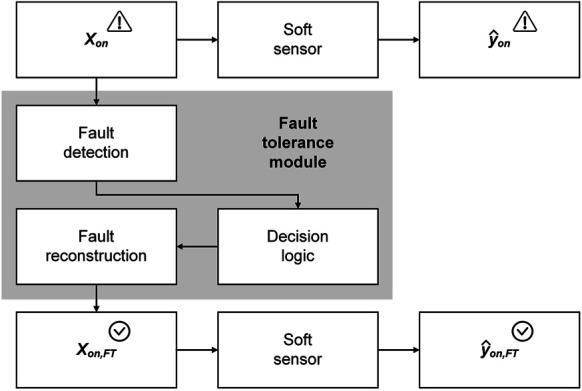
Concept of a fault tolerance module for creating a fault-tolerant soft sensor. When a fault occurs in one or multiple variables of the soft sensor inputs $X_{\mathrm{on}}$, usually the soft sensor prediction ${\hat{y}}_{\mathrm{on}}$ is also faulty dependent on the degree of influence of the variable(s) in the soft sensor model (upper branch). Fault-tolerant soft sensors are capable of compensating for faults in the inputs by the following procedure: Initially, the sensor fault is detected. Then, a decision logic determines whether or not to reconstruct the faulty sensor reading. This reconstruction results in a fault-free substitute for $X_{\mathrm{on}}$, namely, the fault-tolerant inputs $X_{\mathrm{on},\mathrm{FT}}$. The soft sensor uses these inputs for the determination of a fault-tolerant output (prediction) ${\hat{y}}_{\mathrm{on},\mathrm{FT}}$.

In the last sections, three different approaches to sensor fault detection were described. In the absence of sensor faults, the model input to soft sensors is considered reliable (online validation). But what is the use of online validation if the fault detected in the input data makes the soft sensor prediction unreliable? We know that something is going wrong, but we cannot change anything (upper branch in Figure 5). This is where the fault tolerance of soft sensors comes into play.

In general, modules for fault tolerance can be implemented at two layers of a soft sensor: at the inputs or in the actual soft sensor model.

The first variant of fault-tolerant soft sensors is shown in Figure 5. Here, sensor faults are first detected and, after a decision logic, they are compensated for by a reconstruction of the faulty sensor reading. This reconstruction is equivalent to missing data imputation (Dunia et al., 1996). The outputs of the fault tolerance module in Figure 5 are the inputs to the soft sensor. The inputs and outputs of the described fault tolerant soft sensor are hereinafter referred to as $X_{\mathrm{on},\mathrm{FT}}$ and ${\hat{y}}_{\mathrm{on},\mathrm{FT}}$. For bioprocesses, there are, to our knowledge, no studies available that explicitly address the development a fault-tolerant soft sensor based on fault detection and reconstruction. However, some authors have separately described the fault tolerance module shown in Figure 5, as described in the following.

In the already mentioned study by Huang et al. (2002), an AANN was used for sensor fault detection by means of a symptom signal and fault reconstruction in a fermentation process with variable process length and time-variant behavior. Examples of sensor fault reconstruction using AANN in applications other than biotechnology are given in Kramer (1991), Kramer (1992), and Hamidreza et al. (2014). Variable contribution statistics ($T^{2}$ and SPE) in a MSPC model were used by Lawal and Zhang (2017) to detect and subsequently reconstruct faulty sensor reading within a crude distillation unit. In the above-mentioned study by Guo and Nurre (1991) an ANN was used to learn the patterns of both fault-free and faulty sensor readings to detect sensor faults. A separate ANN was trained to reconstruct the faulty readings.

In the second variant of fault-tolerant soft sensors, the faulty inputs are not reconstructed but the soft sensor algorithm itself is responsible for the fault management. For bioprocesses, there is, to our knowledge, only one study available that explicitly addresses fault tolerance by adapting the soft sensor models (Krause et al., 2015). The already described MSPC model developed by Krause et al. (2015) is capable of giving reliable predictions ${\hat{y}}_{\mathrm{on},\mathrm{FT}}$ (here: higher-level process quantity) in the presence of sensor faults. As mentioned, PSO is used to find the best models with respect to historical process data. When sensor readings in $X_{\mathrm{on}}$ differ significantly from historical data $X_{\mathrm{hist}}$, they are penalized by the PSO cost function. This in turn results in a drastically decreased contribution of the faulty sensor reading and thus a fault-tolerant prediction of ${\hat{y}}_{\mathrm{on}}$. With this approach to sensor fault tolerance the same has to be considered as with the entire MSPC concept: Both are ultimately based purely on a statistically significant deviation from $X_{\mathrm{on}}$ to $X_{\mathrm{hist}}$ and are thus strongly dependent on the size and quality of the process data pool for $X_{\mathrm{hist}}$.

**In summary**, it must be noted that, with very few exceptions, there are no studies on fault-tolerant soft sensors for the process industry. With regard to fault detection before the subsequent reconstruction, all three methods described above are applicable. However, for the methods of variable contribution in a MSPC model and pattern recognition methods, a separate model must be developed to reconstruct the faulty sensor reading. Symptom signal methods offer the advantage that the reconstructed sensor reading is directly available.

## Conclusion

Based on an overview of the status quo of soft sensor development and online implementation, this review article describes the challenges of variable process lengths, multiple phases, and sensor faults, and critically discusses the corresponding solution approaches. The challenges are considered both individually and synchronously, and the solution approaches are evaluated in terms of their practicality and applicability to bioprocesses.

**Variable process lengths**: Data synchronization techniques are employed to ensure that soft sensors provide correct predictions despite variable process lengths. For data synchronization, indicator variable techniques and particularly DTW dominate the bioprocess literature compared to curve registration techniques. Indicator variables alone can only be used for the alignment of the entire process lengths. In contrast, DTW and curve registration techniques can additionally be used for the alignment of landmarks. Indicator variable techniques require a higher degree of process knowledge (selection of appropriate process variables etc.) compared to DTW and curve registration techniques. DTW is the technique of choice when a solution is sought that does not require much process knowledge (compared to indicator variable techniques) and fine-tuning (compared to curve registration techniques).

**Multiple process phases**: The basic strategy for coping with multiple process phases is to divide the process datasets into individual phase segments and develop separate models for these segments. For the detection and division of process phases, trajectory-based and correlation-based methods have been proposed in the literature. Methods based on the progression of process trajectories, most notably via DTW, have to date been proposed more frequently in the bioprocess literature compared to correlation-based methods. Reasons for this include better comprehensibility of algorithms, easier interpretability of results, and coincidence with actual operational process phases in trajectory-based methods (Luo et al., 2016). On the other hand, correlation-based methods offer the advantage that they can be developed almost entirely without process knowledge. The consideration of phase transitions has so far been described only for trajectory-based methods (via FCM; Luo et al., 2016); for correlation-based methods, the consideration of phase transitions is still lacking.

**Sensor faults**: If the input to a soft sensor is faulty, there is a high probability that the output is faulty as well. Despite this obvious relation, studies on the detection of or even tolerance to sensor faults in bioprocesses are rare. Methods based on variable contributions in MSPC models are well established in the process industry for the identification of sensor faults. Further research is required to evaluate the applicability of these methods to highly collinear bioprocesses, as groups of correlating variables are often displayed as faulty due to smearing effects (van den Kerkhof et al., 2013). Symptom signal methods have been used to detect sensor faults and to reconstruct faults in bioprocesses. These methods, especially AANN, seem to be promising tools for the fault tolerance of soft sensors. The recognition of previously trained fault patterns has been used in mechanical engineering for fault detection, but to our knowledge has not yet been addressed in the bioprocess field. However, it can be assumed that this branch of machine learning will also increase in popularity in the field of bioprocesses due to steadily growing libraries of ready-to-use algorithms. For all three approaches presented for the detection of sensor faults (symptom signal, MSPC, pattern recognition) it could be shown that they are also capable of detecting simultaneously occurring sensor faults.

**Synchronous consideration of the three challenges**: The development of soft sensors for bioprocesses with multiple phases and variable process lengths has been investigated in several studies (e.g., Ündey et al., 2003; Luo et al., 2016). As described above, landmark-based data synchronization is particularly suitable for multiphase processes. For sensor fault detection for bioprocesses with variable lengths but without multiple phases individual studies exist (Krause et al., 2015; Brunner et al., 2019). Regarding sensor fault detection for multiphase bioprocesses with variable lengths, the question remains open as to which of the three methods presented is most suitable. This is because there is to the best of our knowledge only one study that provides a solution for the synchronous occurrence of all three challenges for bioprocesses (Huang et al., 2002).

The core conclusions of this review article are as follows:

• The choice of methods to handle variable process lengths and multiple process phases is dependent on the level of implementable process knowledge.
• The dilemma with sensor fault detection via soft sensors is that the input to the soft sensor can itself be erroneous.
• There is a clear research gap regarding the validation of the input data to soft sensors.
• Specifically, approaches to the tolerance of soft sensors to sensor faults need to be found.

Closing these gaps not only will allow existing sensor networks to be used more efficiently to monitor bioprocesses but will also strengthen confidence in soft sensors and PAT.

## Glossary

b: model coefficients
E: process end
e: residual between prediction and original sensor reading
h: warping function
i: index for sensor of interest
j: number of process variables
L: landmark
m…n: indices for sensors other than sensor i
$r_{i}$: reliability of sensor i
SPE: squared prediction error (sometimes denoted as Q)
t: time
X: process data = input to soft sensor
$X_{\mathrm{hist}}$: historical process data
$X_{\mathrm{on}}$: online process data
$X_{\mathrm{on},\mathrm{FT}}$: fault-tolerant online process data
$X_{\mathrm{sync}}$: synchronized process data
y or Y: target quantity (vector or matrix) = output of soft sensor
$y_{\mathrm{on}}$: online data of target quantity
$y_{\mathrm{hist}}$: historical data of target quantity
${\hat{y}}_{\mathrm{hist}}$: prediction for historical data of target quantity
${\hat{y}}_{\mathrm{on}}$ or ${\hat{Y}}_{\mathrm{on}}$: prediction for target quantity (vector or matrix)
${\hat{y}}_{\mathrm{on},\mathrm{FT}}$: fault-tolerant prediction for target quantity
φ: combined fault detection index
**AANN**: autoassociative neural network
**ANN**: artificial neural network
**COW**: correlation optimized warping
**CPP**: critical process parameter
**CQA**: critical quality attribute
**CSTR**: continuous stirred-tank reactor
**(D/M)DTW**: (derivative/multivariate) dynamic time warping
**DSP**: downstream processing
**EC**: European Commission
**FCM**: fuzzy c-means
**FDA**: Food and Drug Administration
**GMM**: Gaussian mixture model
**ISPE**: International Society for Pharmaceutical Engineering
**JIT**: just-in-time
**MLR**: multiple linear regression
**MSPC**: multivariate statistical process control
**OECD**: Organisation for Economic Co-operation and Development
**OPC (UA)**: open platform communications (unified architecture)
**PSO**: particle swarm optimization
**RBC**: reconstruction-based contributions
**RGTW**: relaxed-greedy time warping
**PAT**: process analytical technology
**PCA**: principal component analysis
**PCR**: principal component regression
**PLS(R)**: partial least squares (regression)
**QbD**: quality by design
**SCADA**: supervisory control and data acquisition
**SVR**: support vector regression
**USP**: upstream processing
**VIP**: variable importance in the projection
**XTW**: extrapolative time warping
